# Targeting Neutrophil Adhesive Events to Address Vaso-Occlusive Crisis in Sickle Cell Patients

**DOI:** 10.3389/fimmu.2021.663886

**Published:** 2021-04-28

**Authors:** Vasilios A. Morikis, Alfredo A. Hernandez, John L. Magnani, Markus Sperandio, Scott I. Simon

**Affiliations:** ^1^ Department of Biomedical Engineering, University of California-Davis, Davis, CA, United States; ^2^ GlycoMimetics Inc., Rockville, MD, United States; ^3^ Institute for Cardiovascular Physiology and Pathophysiology, Walter Brendel Center for Experimental Medicine Biomedical Center, Ludwig Maximilians University, Walter Brendel Center, Munich, Germany

**Keywords:** vaso-occlusion crises, neutrophil, selectin, integrins, sickle cell disease

## Abstract

Neutrophils are essential to protect the host against invading pathogens but can promote disease progression in sickle cell disease (SCD) by becoming adherent to inflamed microvascular networks in peripheral tissue throughout the body. During the inflammatory response, leukocytes extravasate from the bloodstream using selectin adhesion molecules and migrate to sites of tissue insult through activation of integrins that are essential for combating pathogens. However, during vaso-occlusion associated with SCD, neutrophils are activated during tethering and rolling on selectins upregulated on activated endothelium that line blood vessels. Recently, we reported that recognition of sLe^x^ on L-selectin by E-selectin during neutrophil rolling initiates shear force resistant catch-bonds that facilitate tethering to endothelium and activation of integrin bond clusters that anchor cells to the vessel wall. Evidence indicates that blocking this important signaling cascade prevents the congestion and ischemia in microvasculature that occurs from neutrophil capture of sickled red blood cells, which are normally deformable ellipses that flow easily through small blood vessels. Two recently completed clinical trials of therapies targeting selectins and their effect on neutrophil activation in small blood vessels reveal the importance of mechanoregulation that in health is an immune adaption facilitating rapid and proportional leukocyte adhesion, while sustaining tissue perfusion. We provide a timely perspective on the mechanism underlying vaso-occlusive crisis (VOC) with a focus on new drugs that target selectin mediated integrin adhesive bond formation.

## Introduction

Sickle cell disease (SCD) is an inherited disease characterized by defects in hemoglobin that distorts red blood cells (RBC) into a “sickle” shape (1–3). Clinical manifestations common to this inherited hemoglobinopathy, chronic hemolysis, and erythrocyte adhesion that can lead to vaso-occlusion (4). Vaso-occlusive crisis (VOC) is defined by clinical episodes of severe ischemic pain due to lack of blood flow resulting in cumulative and irreversible organ damage. As such, the occurrence of VOC is a serious clinical event that is responsible for the increased morbidity and mortality of sickle cell patients. The clinical sequalae associated with VOC often manifests in episodes of pain that vary in intensity and duration as a function of patient comorbidities such as diabetes. Some patients experience only infrequent pain crises each year, while others require acute hospitalization for treatment. Pain management during VOC often includes the prescription of opioids that, while effectively diminishing pain, do not treat the root cause and therefore do not significantly lower the risk of a catastrophic clinical event (5). Thus, treatments to prevent genesis of VOC as opposed to pain management is a primary challenge for scientists aiming to establish effective treatments that target the initiation and block progression (6). In this perspective we review the recent studies highlighting the importance of neutrophil inflammatory recruitment and the role of selectin and integrin adhesive receptors in progression to clinical crisis. We endeavor to illuminate the lessons learned from preclinical studies in mouse models of SCD and how they apply to human disease in order to evaluate the outcome of recent clinical trials that target neutrophil recruitment.

**Figure 1:**
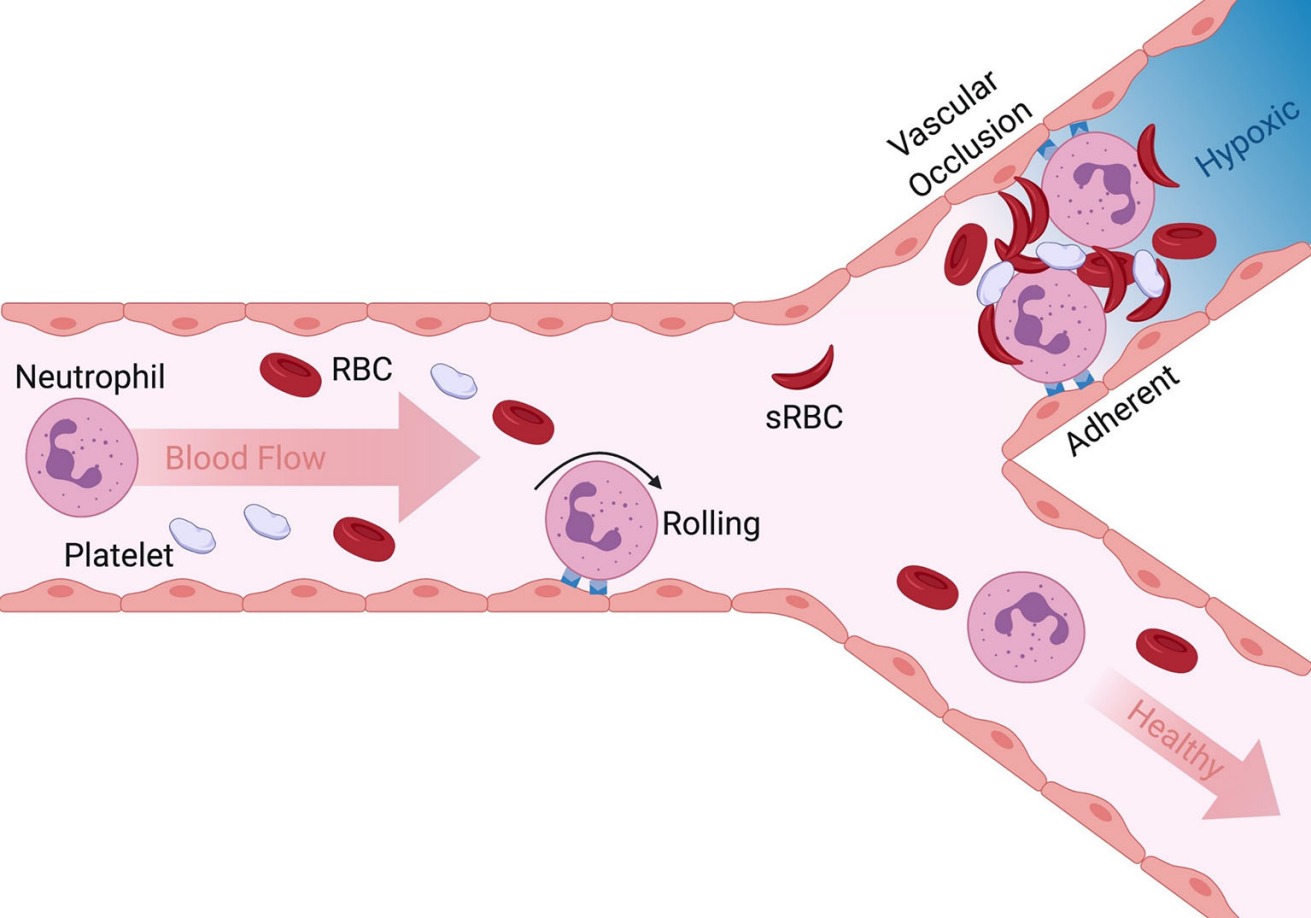
Overview of initiation and progression of VOC in SCD. Neutrophil rolling to arrest on inflamed endothelium is a normal innate immune response to tissue injury. Adherent neutrophils can also capture sickle red blood cells and platelets in post capillary venules leading to vascular occlusion (upper bifurcation). Dramatic reduction in blood flow and lack of oxygen results in hypoxia, episodic painful and often leads to organ failure. When inappropriate neutrophil activation and adhesion is absent and secondary capture of RBC and platelets limited, the vasculature remains perfused and healthy (lower pathway).

The etiology of VOC involves the innate immune response that enables white blood cells (WBC) predominantly neutrophils to capture on adhesive receptors upregulated on inflamed endothelium. Captured neutrophils become activated during interaction with endothelium at sites of inflamed microvasculature leading to upregulation of additional adhesion molecules on the plasma membrane that serve to recruit additional circulating cells, such as sickle RBC (sRBC) and platelets that aggregate causing rapid occlusion of the microvasculature leading to vessel ischemia (**Figure 1**) (2, 7, 8). Contributing to microvascular occlusion is a greater capacity for neutrophil extracellular trap (NET) formation characterized by decondensed chromatin enriched in citrullinated histones and bound to granular enzymes (9). The pathophysiology and requirement for pain management directly associated with VOC in patients has been extensively reviewed (2, 4, 6, 10, 11). As such, the focus of this review is to highlight the role of neutrophils in the initiation and propagation of VOC, as well as targeted pharmacological interventions against adhesion molecules critical for neutrophil recruitment and heterotypic adhesion with other blood cells during flow within vessels.

Neutrophil capture on inflamed endothelium is initiated by Sialyl Lewis^x^ (sLe^x^) bearing selectin ligands on cells in flow (12–15). Ligand recognition by selectins is an early step in the inflammatory process mediating leukocyte rolling and platelet capture, as such selectins are a primary target for therapeutics to prevent propagation of VOC. Early studies on the specific role of cellular adhesion molecules that induce VOC were carried out in mice due to the availability of genetic knockout models of WBC adhesion *via* selectins and integrins. However, human and mouse genomes have diverged in the past 75 million years and as such selectin function differs between the mouse and human innate immune response (**Table 1**). While selectin targeting has been a major focus in designing VOC treatments, there is a need to better understand how selectins participate in precipitating neutrophil recruitment leading to VOC in more accurate models of human SCD in order to design informed therapeutic treatments.

**Table 1:** Differences between mouse and human selectins.

	Human *versus* Mouse
**E-selectin**	Lectin domain homology: 72% (16)
	EGF domain homology 60% (17)
	Mice have a greater interdomain angle (more flexibility, better binding to sLex) (17)
	ESL-1 is not a functional E-selectin ligand in humans (18)
**P-selectin**	TNFa, IL1B and LPS increase P-selectin mRNA in mice but decrease it in humans (19)
**L-selectin**	Human E-selectin binds sLex on L-selectin (mouse L-selectin lacks fucosylation) (18)
	Fucosyltransferase 9 (FUT9) plays a key role in human L-selectin biosynthesis (20)

## Selectin Function and Bond Mechanics in Vaso-Occlusive Crisis in Humans

**Figure 2:**
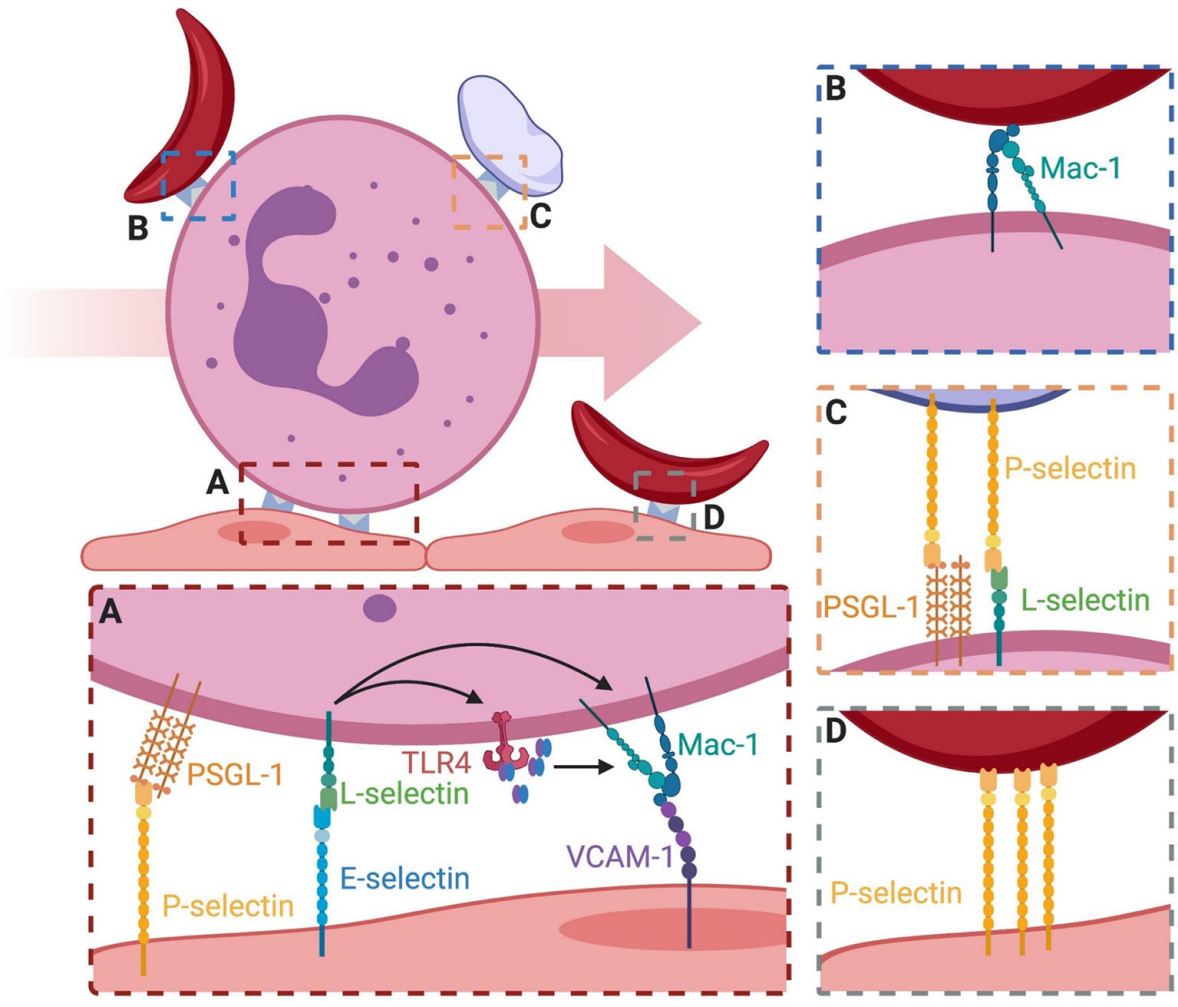
Binding events that lead to vasculature occlusion on inflamed endothelium. **(A)** Neutrophil rolling is mediated by P-selectin and E-selectin binding PSGL-1 and L-selectin. Signaling through E-selectin/L-selectin bonds results in (1) release of MRP8/14 and endogenous activation of surface TLR4 which primes integrin for activation and (2) direct signaling of transition to a high-affinity integrin state capable of binding ligand. High affinity integrin is capable of stable bond formation to VCAM-1 and ICAM-1 on endothelial to mediate neutrophil arrest. **(B)** Secondary capture of sRBC to adherent neutrophils is mediated *via* Mac-1. The mechanism of capture is an active area of research. **(C)** Secondary capture of platelets to adherent neutrophils is mediated through platelet surface P-selectin and its binding to PSGL-1 and L-selectin. **(D)** Direct engagement of sRBC to the surface of endothelial cells has been attributed to P-selectin, similar to secondary capture *via* Mac-1.

To understand the etiology of VOC in terms of the initiation and development of a congested microvascular bed, selectin function during neutrophil recruitment must be examined. In humans three selectins participate in forming stable but transient adhesive bonds with carbohydrate structures between cells in interactions characterized by fast kinetics that allow cell adhesion to proceed under the shear forces of blood flow (21, 22). Expression of endothelial E-selectin requires *de novo* protein synthesis, and is upregulated within hours of cytokine activation at inflamed sites experiencing disturbed blood flow or focal tissue insult (23, 24). P-selectin is preformed and stored in Weibel Palade Bodies (WPB) of endothelial cells and in α-granules of circulating platelets and is rapidly mobilized from these storage sites by merging with the plasma membrane where it participates in tethering and rolling of leukocytes and platelets on inflamed endothelium (25, 26). Leukocyte expressed L-selectin is a glycoprotein that not only binds sLe^x^ as its primary carbohydrate recognition motif expressed by PSGL-1 on neutrophils, but it also presents sLe^x^ to facilitate neutrophil homing and subsequent activation within inflamed venules (27, 28). E-selectin binding to sLe^x^ supports capture and rolling of human, but not murine neutrophils thereby providing a key event for subsequent mechanosignaling of integrin activation that mediates leukocyte arrest even in absence of chemokine signaling (29). Neutrophil homotypic adhesion is observed as secondary capture of a neutrophil from the blood stream by a rolling or arrested neutrophil *via* L-selectin binding of PSGL-1 between cells (**Figure 2A**) (29–31). While E-selectin and P-selectin both function in the early capture and adhesion of leukocytes to the vascular endothelium, there are some distinct differences. All selectins share a similar structure characterized by a lectin binding domain, epidermal growth factor domain, a variable number of short consensus repeats (9 for P-selectin, 6 for E-selectin, and 2 for L-selectin), a transmembrane domain, and a cytosolic tail (13). Despite the similarities in structure, the binding kinetics and capacity to form durable bonds that mechanotransduce activation of integrins are quite different (22, 32). P-selectin projects the furthest above the endothelial surface and is thought to provide the initial interaction between leukocytes in the free stream through recognition of PSGL-1, although it also can bind additional ligands including sulfated polysaccharides (33). However, P-selectin does not mechanotransduce activation of integrin on bound leukocytes in the same manner as E-selectin binding to L-selectin. E-selectin forms longer-lived shear resistant bonds with L-selectin compared with P-selectin, that is independent of recognition of its other cognate ligands (i.e. PSGL-1, CD44). L-selectin appears to be unique on human neutrophils due to its recognition by E-selectin and capacity to actively condense into bond clusters that mechanotransduce signals leading to β_2_-integrin activation and adhesion (29). This function is attributed to E-selectin dependent formation of a high-affinity complex with sLe^x^ under precise hydrodynamic conditions to form a catch-bond with L-selectin (34). A molecular model of E-selectin binding sLe^x^ under tension predicts that it adopts an extended conformation, which was verified by small-angle X-ray scattering of it bound to purified ligand (34). Extended E-selectin/sLe^x^ complexes form shear resistant bonds characterized by adhesive lifetimes that are long lived (~ 500 msec) at sufficiently high hydrodynamic shear stress. Whereas at low shear rates (i.e. < 1 dyne/cm^2^) associated with microvascular regions of slow blood flow and ischemia, adhesive bonds are less durable and dissociate more rapidly. This phenomenon is denoted catch-bond behavior (34–38). E-selectin binding to sLe^x^ results in co-localization of L-selectin and PSGL-1 on the trailing edge of neutrophils, a process associated with β_2_-integrin activation and neutrophil arrest (26, 29). A stepwise process depicted in **Figure 2** illustrates how neutrophils tethered on endothelial P-selectin, may facilitate E-selectin and L-selectin to engage and mechanically signal cell activation and arrest. Selectins are a prime target for inhibition of the inflammatory response that initiates VOC, with the rationale that blocking the adhesive steps in the vaso-occlusive process will prevent propagation of congestion and decrease the duration and intensity of painful VOC in patients with SCD.^2^

The importance of selectins in VOC was rigorously studied by Hidalgo et. al., who first reported in a humanized sickle cell mouse model that E-selectin mediated activation of β_2_-integrins at the leading edge of a neutrophil promotes transition from rolling to arrest in an inflamed microvascular bed (39). In this model of acute lethal VOC, a proinflammatory state was induced in post capillary venules by injection of tumor necrosis factor-α (TNF-α). This resulted in capture of sRBC by activated α_M_β_2_ integrin (macrophage-1 antigen, Mac-1) clustered on the leading edge of arrested neutrophils. Genetic deletion of E-selectin (Sele^-/-^) or P-selectin (Selp^-/-^) provided equivalent inhibition of neutrophil capture and reduced RBC/WBC aggregates at early time points within 3 hours of TNF-α stimulation. However, E-selectin knockouts exhibited reduced neutrophil arrest and RBC/WBC aggregates were undetected at later time points of 3-5 hours post stimulation. In contrast, P-selectin knockouts had a spike in the occurrence of RBC/WBC aggregates at 3-5 hours post TNF-α stimulation, resulting in VOC equivalent to control WT mice experiencing a VOC. Significant was the observation that E-selectin knockout mice maintained low levels of secondary capture of RBC that maintained higher rates of blood flow (39). One may conclude that inhibition of P-selectin effectively delayed VOC progression within the initial hours of an inflammatory insult, however, the presence of E-selectin that maintains expression on endothelium elicits sustained neutrophil-RBC recruitment that is responsible for long term VOC. Of note, another important difference between the human and the mouse system concerns the transcriptional regulation of P-selectin. While stimulation of endothelial cells with the proinflammatory cytokine TNF-α leads to the transcriptional upregulation of endothelial P-selectin in the mouse system, endothelial P-selectin expression is downregulated following stimulation with TNF-α in human microvasculature. This has been elegantly demonstrated in the TNF- α stimulated cremaster muscle of mice lacking mouse P-selectin, but expressing human P-selectin supporting the concept that P-selectin in humans might be less important in the pathogenesis of VOC progression than E-selectin (19).

E-selectin mediates recruitment by engaging L-selectin on human neutrophils during rolling which results in arrest *via* activation of LFA-1 to bind with ICAM-1 expressed on inflamed endothelium (29). Secondary capture of RBCs can also be observed in parallel plate flow channels that mimic the hydrodynamic conditions observed in normal versus inflamed microcirculation. In such a model, it was confirmed that human neutrophil capture and RBC/WBC heterotypic aggregates are enhanced in sickle cell patients (**Figure 2B**) (40). It is noteworthy that antibodies that block E-selectin mediated activation of Mac-1 on neutrophils, also blocks heterotypic RBC/WBC aggregation. This directly correlates with increased survival compared to isotype nonblocking control antibody (41). Platelet/WBC aggregates are also significantly reduced in Mac-1 knockout mice during an induced VOC event in mice (41). Thus, both E-selectin and Mac-1 are implicated as primary capture molecules initiating and propagating VOC. However, β_2_-integrins including Mac-1 and LFA-1 are not strategic targets for treatment of VOC in SCD, as leukocytes require these integrins to maintain immune surveillance and to combat infection. Complete blockade of integrin subunit function can be dangerous for high risk immunodeficient patients as realized in clinical trials of the CD11a blocker Efalizumab that was associated with viral meningitis and bacterial sepsis in a subset of patients, which triggered it’s rapid withdrawal from the market in 2009 (42). Another strategy is to target selectins in order to reduce integrin activation and neutrophil capture. In this manner, integrin activation is maintained *via* alternate pathways such as by chemokine receptor ligation, thereby preserving neutrophil and T cell function necessary to combat infections.

There is evidence that soluble E-selectin levels in serum are higher in SCD patients than in healthy subjects and can increase further during acute VOC in SCD patients (43). Kato et al. measured the level of E-selectin, P-selectin, VCAM-1, and ICAM-1 in the plasma of 160 patients suffering from VOC over a four-year period (44). E-selectin, P-selectin, and the integrin ligand VCAM-1 were all significantly increased in patients suffering from SCD compared to controls. A significant finding was that patients with high levels of soluble E-selectin directly correlated with elevated risk of mortality, while those with high levels of soluble P-selectin did not significantly correlate with increased mortality. This implicates neutrophil binding to E-selectin as a primary means of Mac-1 activation and binding to its ligands as a predominant feature that drives neutrophil adhesion and progression of VOC.

E-selectin and P-selectin recognize a variety of ligands on the neutrophil plasma membrane. L-selectin and PSGL-1 are prominent among these since they can function not only to tether neutrophils to inflamed post capillary venules, but allow function as mechanosignaling receptors that activate rapid cell arrest *via* high affinity β_2_-integrin bond formation in shear flow (12). Recruitment of human neutrophils on inflamed endothelium is supported by the preferential recognition of L-selectin by E-selectin and PSGL-1 by P-selectin, respectively (22, 45–47). Studies of neutrophil recruitment using vascular mimetic microfluidic channels under defined hydrodynamic shear on a substrate of recombinant E-selectin revealed that L-selectin and PSGL-1 co-localize into clusters on neutrophil microvilli and spontaneously activate β_2_-integrin bond formation on ICAM-1 (48). Recently, it was reported that formation of catch-bonds between E-selectin and L-selectin effectively induce the extension of β_2_-integrin that supports its binding to ICAM-1 during neutrophil deceleration that can prime the transition to a high-affinity state (29). A mechanism has been identified that involves the calcium-modulating proteins myeloid related protein-8 (MRP8) and MRP14 (also known as MRP8/14, S100A8/S100A9, and calprotectin). MRP8/14 can modulate myeloid cell function by binding Toll-like receptor-4 to induce calcium signaling and cytoskeletal reorganization (49–51). A complex of MRP8/14 released from leukocytes in mouse and human models of SCD are known to contribute to vascular inflammation and tissue injury. Supporting such a mechanism is the observation that MRP8/14-/- mice exhibited reduced neutrophil accumulation and lesion severity, implicating it in regulation of neutrophil recruitment (52). This mechanism is associated with acute inflammation, as neutrophils captured on E-selectin secrete MRP8/14 which then binds TLR4 on the same cell to initiate integrin extension (29, 49). Blocking TLR4 binding by treatment with antibody, or inhibiting E-selectin/L-selectin bond formation under shear by addition of the sLe^x^ mimetic Rivipansel, blocked the release of MRP8/14 and significantly reduced integrin extension and transient bond formation with ICAM-1 (29). These data reveal a pathway by which E-selectin/L-selectin bonds induce the release of MRP8/14 that bind TLR4 and in an autocrine manner signal conformational extension of LFA-1. This along with signaling *via* engaged and clustered L-selectin promotes long-lived high-affinity integrin bonds and the efficient arrest of rolling neutrophils in the microcirculation (22, 45, 53). This early step in the multistep process of neutrophil recruitment facilitates deceleration *via* E-selectin mediated rolling that upregulates high-affinity LFA-1 and subsequently Mac-1. When blocked with small molecule antagonists or anti-L-selectin antibody, LFA-1/Mac-1 mediated neutrophil recruitment and secondary capture observed in VOC is prevented (**Figures 2A, B**).

While E-selectin and P-selectin mediate the capture of individual neutrophils, it is important to highlight heterotypic adhesive interactions in the genesis of VOC. Secondary capture of free-flowing neutrophils, platelets and erythrocytes with adherent WBC are implicated in various disease states such as atherosclerotic plaque formation and VOC (54, 55). Homotypic neutrophil-neutrophil adhesion is mediated by L-selectin on one cell and PSGL-1 on the adjacent cell (**Figures 2B, C**). This results in strings of rolling neutrophils aligned along the direction of shear flow, which is thought to be relevant in the pathogenesis of VOC. Cell aggregation leading to microvascular congestion is also mediated by neutrophil-platelet interactions. Activated platelets express P-selectin and can bind free flowing neutrophils, thereby amplifying the surface area and the likelihood of capture on inflamed endothelium or on adherent neutrophils (**Figure 2C**) (56, 57). Adherent platelets help orchestrate diapedesis of neutrophils by Mac-1 binding to platelet GPIIb/IIIa in a fibrinogen dependent manner (58). Cellular crosstalk between platelet P-selectin binding to neutrophil PSGL-1 and Mac-1/fibrinogen/GPIIb/IIIa supports the efficient trafficking of neutrophils to sites of inflammation. Taken together, this implicates Mac-1 and P-selectin as adhesion receptors that may play a central role in the heterotypic platelet adhesive events that occur downstream of E-selectin in the progression of VOC. While the importance of selectins and their ligands cannot be overstated, there are key differences in function between human and mouse models that must be appreciated in order to efficiently develop effective therapeutics to prevent VOC.

## Of Mice and Men, What Are the Lessons for Treatment of VOC

Much of the knowledge base surrounding E-selectin function stems from transgenic mouse models, which indicate that rolling *via* PSGL-1 elicits a distinct mechanosignal that leads to integrin mediated neutrophil arrest. A key difference in mouse E-selectin ligands versus humans stems from fucosyltransferase-9/7 function, which is critical for assembly of the ligands that decorate glycoproteins supporting neutrophil rolling on E-selectin in human microvasculature, but not mice (**Table 1**) (18–20). These fucosyltransferases function to modify O- and N-linked carbohydrates on L-selectin to express the E-selectin ligand sLe^x^ (18, 20, 59). This adaption in humans enables the sLe^x^ on L-selectin to be recognized by E-selectin directly, which does not occur for mouse L-selectin. The observation that mouse E-selectin plays a critical role in mechanosignaling during rolling in inflamed venules is accounted for by the finding that recognition of sLe^x^ on PSGL-1 results in its co-clustering with L-selectin, which in turn elicits outside-in signaling of integrin activation (27). Knockout mice lacking L-selectin do not possess the capacity for direct outside-in signaling of integrin activation, yet they can still roll *via* endothelial selectins. This difference between human and mouse E-selectin ligands may account for the spontaneous vaso-occlusion observed in humans and not mouse microvasculature (60). It is clear that in humans the E-selectin/L-selectin signaling complex represents a primary step in neutrophil adhesion even in the absence of cytokine or chemokine. Targeting this mechanosignaling pathway by antagonizing selectin catch-bond formation could provide a strategic tipping point for therapeutic targets to prevent development of VOC in SCD patients.

Another difference in the regulation of selectin expression between mouse and humans consists in downregulation of human and upregulation of mouse P-selectin expression during TNF-α stimulated inflammation (19). Evidence supporting a primary role for P-selectin in SCD was demonstrated in a mouse model lacking P-selectin that showed a protective effect in pulmonary VOC (61). To further illuminate the contribution of P-selectin in human VOC, the next logical step would be to generate sickle cell mice lacking mouse P-selectin but edited to express human P-selectin. Such a hybrid mouse line could be used to reveal a species difference in P-selectin function.

## Targeted Therapeutics Against Leukocyte and Platelet Activation in Vaso-Occlusive Crisis

Mouse models of SCD have been indispensable for dissecting the molecular determinants of cytokine driven and selectin mediated vascular congestion, including to test selectin targeted drug treatments (16, 62). In the case of the small molecule sLe^x^ mimetic Rivipansel^®^ (GMI-1070), we tested its capacity to antagonize the rolling and activation of neutrophils in blood over time of treatment in a Phase-3 clinical trial for treatment of VOC (29). Employing real-time immunofluorescence imaging of neutrophils in microfluidic channels, we examined the molecular dynamics of selectin-ligand receptor engagement during the transition from cell tethering and rolling to arrest on recombinant E-selectin and ICAM-1 *ex-vivo*. Rivipansel was found to block E-selectin recognition of sLe^x^ on L-selectin and antagonize the formation of catch-bonds and outside-in signaling of integrin mediated cell arrest in human SCD patient blood. GSnP-6, a novel PSGL-1 mimetic that blocks its recognition by E-selectin and P-selectin, effectively doubled the rolling velocity of neutrophils, but had no effect on L-selectin outside-in signaling of β_2_-integrin or the frequency of neutrophil rolling to arrest (29, 63). This differential glycomimetic inhibition of neutrophil rolling versus outside-in activation of integrins *via* selectins reveals a novel mechanosignaling circuit. E-selectin transmitted tension on L-selectin induces clustering and signaling within sites of focal adhesion, which is markedly different than that observed in mouse neutrophils. PSGL-1 is the primary ligand for E-selectin mediated rolling in mouse as compared with L-selectin on human neutrophils. To determine the therapeutic efficacy of antagonists directed at sLe^x^ recognition by selectins in treatment of VOC, humanized P-selectin mouse models and/or *ex-vivo* assays could be used to quantify MRP8/14 release and TLR4 signaling of integrin activation.

Improving patient quality of life through preventative treatments of VOC occurrence or decreasing the time to crisis resolution are critical goals for long-term care of patients with SCD. Current standards for clinical treatment serve to modify the pathobiology of the disease through blood transfusions and dosing with hydroxyurea which serves to increase circulating Fetal Hemoglobin (HbF) and diminish the polymerization of intracellular sickle cell hemoglobin (HbS) in adults and pediatric populations (59, 60, 63–66). Studies by Frenette et al. demonstrated the importance of elevated plasma heme in a humanized SCD mouse model. In fact, unstimulated neutrophils isolated from SCD patients rapidly begin forming NETs compared to almost no NET formation in age-matched healthy controls, even when under treatment with hydroxyurea (67). This elevated level of heme induces arrested neutrophils to release NETs that have been associated with hypothermia and rapid death. Modulation of plasma heme does alter the NET release and subsequent survival in this mouse model, leaving the underlying issue of the role of elevated neutrophil arrest efficiency and the onset of vaso-occlusion (8, 9). While allogenic hematopoietic stem cell transplants (HSCT) and gene therapies are currently under investigation for their potential long-term curative effects by reversing the defect in hemoglobin function, these have proven to be costly and currently have limited availability for wide spread treatment of SCD.

Activated platelets contribute to vaso-occlusion by participating in intercellular adhesion leading to formation of cell aggregates (6, 10). Pharmacological inhibition of platelet activation has been achieved using receptor antagonists of P2Y12R, the chemoreceptor involved in ADP-stimulated activation of platelets including platelet degranulation (41, 68). Clinical assessment of P2Y12R antagonists including Prasugrel and Ticagrelor have shown limited efficacy in adolescents and young adults with modest reduction of platelet activity and frequency of VOC (69, 70). These interventions have been ineffective in decreasing the frequency of pediatric VOC, pointing to a more significant role for platelet function on the occurrence of VOC in young adults. Directly targeting platelet adhesion through blocking GPIIbIIIa *via* administration of eptifbatide has been evaluated in a small cohort of subjects undergoing VOC (71). Treatment with eptifbatide did not diminish time to VOC resolution, but did successfully inhibit platelet and leukocyte aggregation, release into circulation, as well as diminished surface expression of P-selectin and serum measures of inflammation such as CD40L, sVCAM-1, sICAM-1, sICAM-3, MIP1α, and TNFα (71, 72).

Current reports link activation of the anaphylatoxin complement- C5a that activates neutrophils, platelets and endothelial cells with induction of vaso-occlusion in SCD mice (73). Blocking P-selectin inhibits C5a-induced vaso-occlusion in liver and skin venules of SCD mice. Moreover, blocking receptor binding with antibody targeting the C5a receptor antagonizes upregulation of endothelial adhesion molecules including VCAM-1, ICAM-1, and E-selectin in liver and lungs. It is noteworthy that a P-selectin deficient SCD mouse model revealed that chronic P-selectin deficiency attenuated liver ischemia but did not resolve cellular senescence and reduced epithelial cell proliferation necessary to maintain hepatic homeostasis (74). Complement from human sera has also been shown to stimulate expression of E-selectin in porcine endothelial cells (75). Likewise, the most abundant enzyme of the complement pathway, MASP-1 induced expression of TNFα, and upregulation of E-selectin on microvascular endothelium (76). Complement activation leads to venous thrombosis, an alternate mechanism driving vaso- occlusion. Interestingly, E-selectin plays a dominant role in venous thrombosis and a specific and potent antagonist of E-selectin adhesive function (GMI-1271; Uproleselan) blocks venous thrombosis, without the need to infuse anti-coagulants such as heparin (77, 78). In addition, blocking E-selectin with Uproleselan also inhibited deep venous thrombosis in a non-human primate model and showed efficacy in treating 2 patients in a case study of calf vein thrombosis (77, 79). Taken together, it appears that complement activation is a viable target to alleviate acute P-selectin and E-selectin dependent mechanisms of microvasculature inflammation that include thrombosis, but may not provide a long-term solution in context of preserving vital organ function.

Numerous anti-adhesive therapies that target RBC-endothelial and leukocyte-endothelial interactions have been completed or are undergoing clinical trials (**Table 2**). Of these, selectin mediating leukocyte and erythrocyte adhesion to the endothelium are prominent targets to limit vaso-occlusion in SCD patients (80–82). Improvement to time of VOC resolution or delay in first incidence of VOC appears to be a common efficacious event in pharmacological inhibitors of selectin binding, such as Rivipansel (5, 83), unfractionated heparin (84, 85), intravenous immunoglobulin (86, 87), and Crizanlizumab (88). Of these, Crizanlizumab^®^ which inhibits P-selectin binding to PSGL-1 currently has completed clinical trials in adults and has the most ongoing trials to assess efficacy in pediatric populations as well as subjects with non-HbSS genotypes (**Table 2**). High dose (5 mg/mL) Crizanlizumab resulted in significant diminishment of the rate of crisis per year and delayed mean time to first or secondary crisis in adults (88). There was, however, no detectable difference in cell hemolysis suggesting that treatment with Crizanlizumab only serves to modify cell adhesion, but not to completely diminish patient systemic inflammation. Antagonism of E-selectin for treatment of VOC using the small molecule Rivipansel did not initially meet the Phase III clinical endpoint of time to readiness for patient discharge. However, subsequent analysis revealed that Rivipansel was efficacious in a subset of patients who were treated earlier from the time of onset of their VOC, with statistical significance demonstrated in both adults and adolescents who received treatment within 26 hours of the initial painful episode of VOC (median difference of 58 hours between Rivipansel and placebo; HR = 0.58; p = 0.03). Importantly, rapid and sustained statistically significant reductions in soluble E-selectin in circulation in this acute setting was observed in the Phase III trial supporting the biological effect of Rivipansel. This raises an important point on the beneficial effects of administering treatment to VOC early in progression and following systemic markers of the capacity of neutrophils to participate in adhesion. Clinical trials on the effects of anti-inflammatory agents such as statins and leukotriene and adenosine receptor inhibitors on resolution of VOC have also been performed (82, 89). Clinical trials assessing the efficacy of simvastatin in adolescents and young adults appear to have a significant effect on occurrence of VOC, as well as a reduction in patient reported pain, and decreased nitric oxide metabolites, human serum C-reactive protein, and soluble cell adhesion molecules ICAM1, VCAM-1, and E-Selectin (89). The data collected over the course of these clinical trials indicates that use of monotherapies targeting selectin expression, activity, and shedding on activated platelet and leukocytes are likely to improve patient quality of life.

**Table 2:** Current agents being investigated to treat VOC.

General Cascade	VOC Prevention Therapeutics	Trials	Targets/Response	Patient Demographic	Results	Dose	Reference
anti-platelet/anti-coagulation	Prasugrel	DOVE (Terminated-lack of efficacy)	P2Y12 receptor antagonist-inhibits adenosine diphosphate (ADP)-mediated platelet adhesion and aggregation	Children 2-17 years old with history of HbSS or HBSβ thalassemia	No significant reduction in the rate of VOC in treatment vs placebo in aggregate. Children 12-17 experienced significant reduction in VOC	Initial dose 0.06 mg/kg with adjusted dosing of 0.04 - 0.12 mg/kg to a maximum of 10 mg	(65)
	Ticagrelor	HESTIA2 (Adults-completed); HESTIA3 (Pediatric-Ongoing)	P2Y12 receptor antagonist-inhibits adenosine diphosphate (ADP)-mediated platelet adhesion and aggregation	Adults 18-30 years, Children 2-18 years	HESTIA2- no reduction in number of days without pain; HESTIA3- not yet reported	Twice daily dose @ 10 to 45 mg (Adults)	(66)
	Epitfbatide	NCT00834899 (Terminated-Low accural, lack of funding)	Synthetic peptide inhibitor of αIIbβ3	Adults 18-55	No significant reduction in median times of discharge or median time of VOC resolution. Lowered serum levels of CD40L, MIP1a, TNFa, and Myoglobin; increases in MMP2/9 and leptin. Transient effect seen post treatment	2 bolus of 180ug/mL 10 min apart, then continous infusion at 2 ug/kg/min for 6 hours	(67, 68)
	Apixaban	NCT02179177 (Terminated-funding exhausted)	Factor Xa Inhibitor	Adults ~30 years	No considerable effect over placebo	2.5 mg/kg twice daily for 6 months	(70, 71)
	Rivaroxaban	NCT02072668 (Ongoing)	Factor Xa Inhibitor	Adults ~30 years	Not yet reported	20 mg daily for 4 weeks	(70, 71)
RBC and WBC adhesion	Crizanlizumab	SUSTAIN (completed); Ongoing: STAND, SOLACE-adults, SOLACE-kids, STEADFAST, SPARTAN, ADORE	Humanized monoclonal (IgG2κ) to P-selectin. Inhibits interaction with P-selectin glycoprotein ligand-1 (PSGL-1)	Various ages from adolescent to adult	Reduction in the duration of VOC	Effective dose of 5mg/kg	(77)
	Rivipansel	Phase 3, Multiceneter, Randomized, Double-Blind, Placebo-Controlled, Parallel-Group Study to evaluate the efficacy and Safety of Rivipansel (GMI-1070) in the Treatment of Vaso-Occlusive Crisis in Hospitalized Subjects with Sicke Cell Disease [NTC02187003 (Completed)]	sLex mimetic; pan-selectin antagonist	Various ages from adolescent to adult (345 participants)	Primary endpoint not met. Ad hoc analysis, treatment within 26 hours met endpoint. Significant reduction in readiness for discharge, 58 hours, median, p = 0.03	Initial loading dose of 20 mg/kg, following ~14 subsequent 10mg/kg doses for 12 hours to achieve minimum plasma levels	(5, 72)
	Intravenous Immunoglobulin (IVIG)	NTC01757418(Ongoing)	Inhibition of RBC-Neutrophil interactions and neutrophil-endothelial interactions	12-65 (Phase 1); 8-14 (Phase 2)	Not yet reported	Intravenous IgG at 800mg/kg (Phase 1); 400mg/kg (Phase 2) at time of crisis	(75, 76)
	Unfractionated Heparin	NTC02098993 (Terminated-Poor enrollment)	Inhibition of P-selectin mediated adhesion	Adults ~30 years	Reduction in the duration of VOC	Initial bolus of 80U/kg followed by 18U/hr IV for 7 days until discharge	(73, 74)
	Propanolol	NCT02012777 (Terminated-lack of results); NCT01077921 (Completed)	Competitive inhibition of βe1-adrenergic receptors; inhibition of RBC binding to the endothelium	Adults ~30 years	Trend in diminished expression of E-selectin, P-selectin, ICAM-1, and VCAM-1. Diminished binding of sickle RBC to the endothelium (ex-vivo)	Standard dose of 40mg every 12 hours for 6 weeks	(56)
Anti-inflammatories	Simvastatin	NCT01702246 (Completed)	HMG-CoA reductase inhibitors, restores eNOS production and reduces CAM expression	Adolescents/ Young Adults ~ 18 years	Significant reduction in pain. Reduction of serum NOx metabolites, hs-CRP, sVCAM-1, sICAM-1, sICAM-3, (s)E-selectin, VEGF	Single oral dose 40mg (>60kg); 30mg (46-60kg), 25mg (35-44kg) daily for 3 months	(78)
	Zileuton	NCT01136941 (Completed)	5-Lipoxigenase inhibitor, inhibits formation of LTB4, LTC4, LTD4	Young adults 12-18 years	No results reported	Initial dosing of 2.4gm/day increased to 3.0 gm/day for 6 weeks	(71)
	Regadenson	NCT01788631 (Ongoing)	Adenosine A2A Receptor (A2AR) Agonist	Adults ∼ 25 years	No reduction in iNKT cell activation, no significant reduction in hospital stay, opiod use, or reported pain.	1.44 mcg/kg/hour over 48 hours	(71)

## Conclusions

Recurrent painful episodes of VOC severely diminish patient’s quality of life and frequently increase the risk of infections and complication of comorbidities that require emergency intervention. There is a need for therapeutics that target the earliest events leading to VOC progression before development of painful crises and end organ damage that typically call for immediate treatment of pain perception rather than the cause of its onset. Neutrophil rolling on E-selectin expressed in the inflamed microvasculature of patients with SCD can initiate integrin activation and firm arrest. Secondary capture of sRBCs and platelet aggregation leads to vascular congestion and tissue ischemia. Targeting these earliest selectin-dependent events in neutrophil recruitment with antagonists that block both initial tethering and signaling of adhesion appear to be clinically effective at mitigating VOC and painful episodes. First clinical trials are promising and should be accompanied by further experimental studies including novel appropriate murine *in vivo* models which address the various differences in the regulation of selectin function between mouse and human and *ex-vivo* analysis of treatment efficacy in inflammatory recruitment especially of neutrophils and sRBC over the course of the disease. Combined, this will shed new light on the role of selectins in the pathogenesis of VOC and hopefully contribute to establishing and refining a potentially beneficial selectin blocking strategy for the treatment of patients suffering from sickle cell disease.

## Author Contributions

VM contributed his knowledge on selectin function in humans, helped outline and wrote the first draft of the manuscript. AH contributed his knowledge on drug efficacy in treatment of VOC in SCD and wrote corresponding sections. MS and JM contributed their vast knowledge of selectin function and clinical mechanisms of VOC and wrote the corresponding sections. SS contributed his vast knowledge on selectin function and neutrophil recruitment in human and mouse inflammation, outlined the manuscript, and wrote corresponding sections. All authors contributed to the article and approved the submitted version.

## Funding

The work was supported by National Institute of Health grants AI047294 (SIS), AI103687 (SIS), and by the German research foundation collaborative research center (SFB), project B01 (MS).

## Conflict of Interest

JM is Senior Vice-President, for Research and Chief Scientific Officer of GlycoMimetics, Inc. He has a financial interest in clinical development of selectin antagonist currently in clinical trials.

The remaining authors declare that the research was conducted in the absence of any commercial or financial relationships that could be construed as a potential conflict of interest.
